# Real‐world assessment of the patient profile, clinical characteristics, treatment patterns, and outcomes associated with erythropoietic and X‐linked protoporphyria

**DOI:** 10.1111/1346-8138.17607

**Published:** 2025-01-06

**Authors:** Samuel M. Silver, Katherine Houghton, Abby Hitchens, Valérie Derrien Ansquer, Malgorzata Ciepielewska

**Affiliations:** ^1^ University of Michigan Medical School Ann Arbor Michigan USA; ^2^ RTI Health Solutions Manchester UK; ^3^ RTI Health Solutions Research Triangle Park North Carolina USA; ^4^ RTI Health Solutions Lyon France; ^5^ Mitsubishi Tanabe Pharma America, Inc. Jersey City New Jersey USA

**Keywords:** erythropoietic protoporphyria, medical records, real world, retrospective, X‐linked protoporphyria

## Abstract

Erythropoietic protoporphyria (EPP) and X‐linked protoporphyria (XLP) are rare genetic disorders. There are limited data regarding how these disorders are managed in real‐world settings. The aim of this study was to document the characteristics and treatment patterns among patients diagnosed with EPP or XLP in general real‐world settings in the United States. We, therefore, conducted a retrospective medical record review of patients diagnosed with EPP or XLP on or before July 1, 2020. Data were analyzed for patients with EPP (*n* = 299) and XLP (*n* = 91). Outcomes included demographic and clinical characteristics, diagnostic testing, therapy recommendations, office visits, emergency department visits, and hospitalizations. Costs were assigned to healthcare resources. Mean (standard deviation [SD]; median) time between the first symptom documented in the medical records and diagnosis was 2.9 (5.1; 1.3) years. The most common pre‐diagnostic tests were liver function, total plasma and erythrocyte protoporphyrin, genetic tests, and renal function. Patients were advised to use sunscreen (85%) or modify their lifestyle (83%). Within 12 months of diagnosis, the mean (SD; median) number of office visits, emergency department visits, and inpatient hospitalizations related to EPP or XLP were 4.0 (3.5; 3.0), 0.8 (1.6; 0), and 0.4 (1.3; 0), respectively. Patients with EPP or XLP have several unmet needs, including timely and accurate diagnosis, symptom relief, and efficacious prevention of phototoxic reactions.

## 1 INTRODUCTION

Erythropoietic protoporphyria (EPP) and X‐linked protoporphyria (XLP) are rare, inherited metabolic disorders. The estimated prevalence of EPP ranges from 1:75000 in the Netherlands to 5:200000 in the United Kingdom (UK).^1^ XLP accounts for approximately 2% and 10% of protoporphyria cases in the UK^2^ and the United States (US),^3^ respectively. These disorders result from alterations in the activities of the ferrochelatase (*FECH*) and aminolevulinic acid synthase‐2 (*ALAS2*) enzymes,^4^, ^5^ causing an accumulation of protoporphyrin in the bone marrow, plasma, and erythrocytes.^6^ When the affected erythrocytes are exposed to visible or long‐wave ultraviolet light, highly oxidized species of oxygen are produced, leading to vascular injuries and release of histamines and chemotactic factors.^6^, ^7^ Patients experience an initial burning or itching feeling (referred to as a prodrome), which can act as warning signals to avoid continued light exposure.^8^ Prolonged exposure causes a painful phototoxic attack that can last for many days and is not responsive to pain medications.^6^, ^9^

For patients with EPP and XLP, there is commonly a long delay to definitive diagnosis,^10^ partially because the conditions may manifest with a broad and non‐specific spectrum of clinical symptoms mimicking other disorders,^11^ and partially because symptoms can be unobservable (i.e., no physical manifestation)^12^ and, therefore, patients may have difficulty communicating their experience. Management of EPP and XLP primarily consists of the prevention of phototoxic reactions by the avoidance of sun exposure following the appearance of prodromal symptoms.^13^ Afamelanotide, a melanocyte‐stimulating hormone analog, administered subcutaneously once every 2 months, has been shown to improve patients’ quality of life, reduce the severity of phototoxic reactions, and increase the amount of time that can be spent in the sun without a phototoxic reaction.^14^, ^15^ Still, afamelanotide is approved only for adults with EPP and is available only from specifically approved centers, which restricts patient access. There is currently no Food and Drug Administration‐approved treatment in the US for patients with XLP or for patients with EPP who are younger than 18 years.

Available information on the diagnosis and management of EPP and XLP primarily comes from research conducted at academic medical centers where physicians have specialized knowledge and access to detailed medical histories.^3^ However, there are few specialists with expertise in these rare disorders. In recognition of this, guidelines were recently published^16^ to standardize care for these disorders. The management and diagnosis of EPP and XLP in general real‐world settings among clinicians with limited experience with these disorders is currently undocumented.

The aim of this study was to document demographic and clinical characteristics, treatment patterns, and related clinical outcomes among patients diagnosed with EPP and XLP in general real‐world settings in the US.

## 2 METHODS

### 2.1 Study design

This study was a retrospective, non‐interventional review of de‐identified medical records of patients diagnosed with EPP or XLP in the US. Healthcare professionals (HCPs) managing patients with EPP or XLP were recruited using a convenience‐sampling approach to abstract data from the medical records of eligible patients. HCPs were asked to self‐confirm diagnosis based on the medical record, but no independent/external review was conducted to confirm the diagnosis. To identify HCPs practicing in more general settings, no quotas based on geographic location, type of practice, or HCP specialty were applied. This study was deemed to be exempt from institutional review board oversight by an RTI Health Solutions International institutional review board.

### 2.2 Study population and measures

Medical records were eligible for abstraction for patients diagnosed with EPP or XLP on or before July 1, 2020.

At the time of EPP or XLP diagnosis, details on sociodemographic characteristics, clinical history, and the diagnostic pathway (specialist referrals and tests conducted) were collected. Data on prescriptions and recommendations for symptom relief were extracted from symptom onset. Information on tests and procedures conducted, office visits and consultations, and specialist referrals within the first year after diagnosis were collected. Data on emergency department (ED) visits and inpatient hospitalizations from symptom onset to 12 months following diagnosis were collected. Costs were derived from standard cost sources based on 2022 US dollars (USD) (Centers for Medicare and Medicaid Services Physician Fee Schedule; Centers for Medicare and Medicaid Services Clinical Laboratory Fee Schedule; IBM Micromedex Red Book; Healthcare Cost and Utilization Project).

### 2.3 Statistical analysis

Data were summarized descriptively. Direct costs were allocated at the patient level (i.e., per‐patient costs) by multiplying the extracted cost of the relevant procedure/test/visit/therapy by the number of units observed for a patient. Mean per‐patient costs were calculated. For the diagnostic pathway, this was based on data that were available in the medical records between the first documented symptom report and diagnosis. Pre‐diagnostic costs included tests for measuring symptoms related to EPP/XLP and referrals to specialists. Data on the number of pre‐diagnostic tests were not collected; thus, the analysis assumed only 1 test per patient. Post‐diagnostic costs included tests for measuring symptoms related to EPP/XLP and specialist referrals within the first 12 months following diagnosis. Prescription therapy and procedure costs included all therapies/procedures from diagnosis to the date of the last available medical record. Costs of prescription therapy were based on the reported dose for the reported frequency. If dose information was missing, the mean observed dose was assumed, except for afamelanotide, where the dose was assumed to be 16 mg if dose information was missing; and hematin, where the dose was assumed to be a 350‐mg vial with 7 mg/mL of hematin. Prescriptions were assumed to be monthly if prescription frequency information was missing, except for afamelanotide, where the frequency was assumed to be every 2 months; hematin, where 1 dose was assumed; and pain relievers, where a 5‐day prescription was assumed. If prescription duration information was missing, the mean observed duration per prescription was assumed. If information on the number of prescriptions was missing, 1 prescription was assumed. Costs of hospitalizations were based on the number of hospitalizations and the duration of each hospitalization reported, and costs of ED visits were based on the number of visits reported.

## 3 RESULTS

### 3.1 Patient characteristics

A total of 386 medical records were abstracted by 136 HCPs (primarily dermatologists [35%], general/family practitioners [18%], and hematologist/oncologists [12%]; Table 1). The majority (94%) of participating HCPs were physicians (Table 1). HCPs reported a diagnosis of EPP for 295 patients (76%), XLP for 87 patients (23%), and both EPP and XLP for 4 patients (1%). Patient sociodemographic characteristics are shown in Table 2.

**TABLE 1:** Healthcare professional characteristics.

	*N* = 136
Profession, *n* (%)	
Physician	128 (94.1)
Nurse practitioner	8 (5.9)
Primary medical specialty, *n* (%)	
Dermatology	47 (34.6)
General practice/family practice/primary care	25 (18.4)
Hematologist/oncologist	16 (11.8)
Pediatrics	12 (8.8)
Gastroenterology	10 (7.4)
Nephrology	8 (5.9)
Internal medicine	7 (5.1)
Hematology	5 (3.7)
Hepatology	5 (3.7)
Genetics	1 (0.7)
Years in practice since full qualification	
Mean (SD)	14.9 (7.7)
Median (Q1, Q3)	14.0 (9.0, 21.0)
Minimum, maximum	2, 35
Primary practice setting, *n* (%)	
Private single‐specialty group practice	49 (36.0)
Private multi‐specialty group practice	30 (22.1)
University/teaching hospital (full time, salaried by hospital)	29 (21.3)
Solo private practice	22 (16.2)
Community hospital practice (full‐time, salaried by hospital)	5 (3.7)
Health maintenance organization	1 (0.7)
Primary practice region, *n* (%)	
Northeast	36 (26.5)
South	36 (26.5)
Midwest	30 (22.1)
West	34 (25.0)

Abbreviations: Q1, first quartile; Q3, third quartile; SD, standard deviation.

**TABLE 2:** Patient characteristics.

	EPP (*n* = 299)^a^	XLP (*n* = 91)^a^
Male, *n* (%)	188 (62.9)	52 (57.1)
White ethnicity, *n* (%)	247 (82.6)	69 (75.8)
Age at first symptom report documented in the medical record (years), mean (SD)	18.6 (16.9)	22.3 (17.4)
Age at diagnosis (years), mean (SD)	23.8 (19.3)	25.9 (18.5)
Region, *n* (%)		
Northeast	75 (25.1)	23 (25.3)
Southeast	58 (19.4)	17 (18.7)
Midwest	72 (24.1)	24 (26.4)
West	70 (23.4)	22 (24.2)
Southwest	19 (6.4)	3 (3.3)
Number of patients for whom a genetic test (*FECH* or *ALAS2*) was conducted, *n* (%)	179 (59.9)	51 (56.0)
Biological relative(s) with known diagnosis of EPP/XLP at time of patient's diagnosis, *n* (%) yes	51 (17.1)	25 (27.5)

Abbreviations: *ALAS2*, aminolevulinic acid synthase‐2; EPP, erythropoietic protoporphyria; *FECH*, ferrochelatase; SD, standard deviation; XLP, X‐linked protoporphyria.

^a^ Includes 4 patients diagnosed with both EPP and XLP.

**FIGURE 1:**
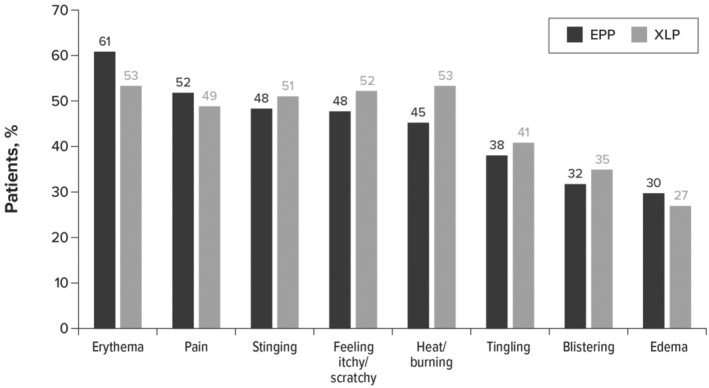
Erythropoietic protoporphyria (EPP) and X‐linked protoporphyria (XLP) symptoms before diagnosis (*n* = 374).

More than half of the patients (*n* = 248; 64%) had at least 1 comorbidity, with the most common being vitamin D deficiency (42%), anxiety (38%), anemia (29%), depression (28%), sleep problems (27%), and iron deficiency (23%). Before receiving a diagnosis, most patients (*n* = 374; 97%) had at least 1 symptom documented (Figure 1).

### 3.2 Diagnostic pathway

**FIGURE 2:**
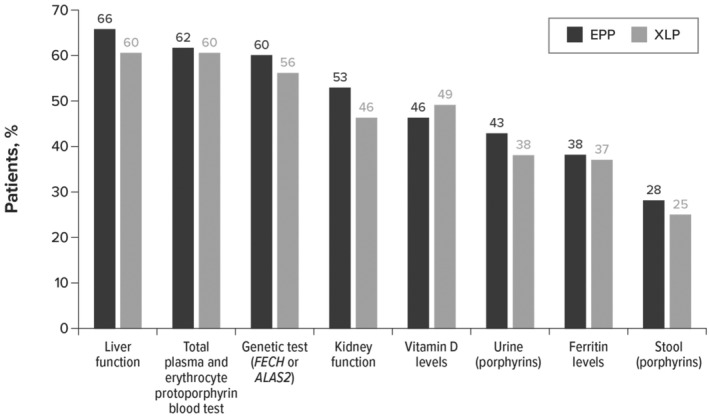
Common pre‐diagnostic tests related to symptoms of erythropoietic protoporphyria (EPP) and X‐linked protoporphyria (XLP) (*n* = 386). *ALAS2*, aminolevulinic acid synthase‐2; *FECH*, ferrochelatase.

The mean (standard deviation [SD]) time between the first symptom documented in the patient medical record and diagnosis was 2.9 (5.1) years, with a median of 1.3 years. Liver function blood tests, total plasma and erythrocyte protoporphyrin blood tests, genetic tests, and renal function blood tests were the most common pre‐diagnostic tests conducted related to symptoms of EPP and XLP (Figure 2).

The mean (SD) number of specialist referrals before diagnosis was 2.8 (3.6) with a median of 2.0. Referrals were primarily to dermatologists (48%) and hematologists (22%). Before diagnosis, patients had received a mean (SD) of 4.4 (2.9) recommendations or therapy orders. Primarily, patients were advised to prevent phototoxic reactions by using sunscreen (82%) or modifying their lifestyle (e.g., avoiding sunlight; 80%).

### 3.3 Prescriptions and recommendations

**FIGURE 3:**
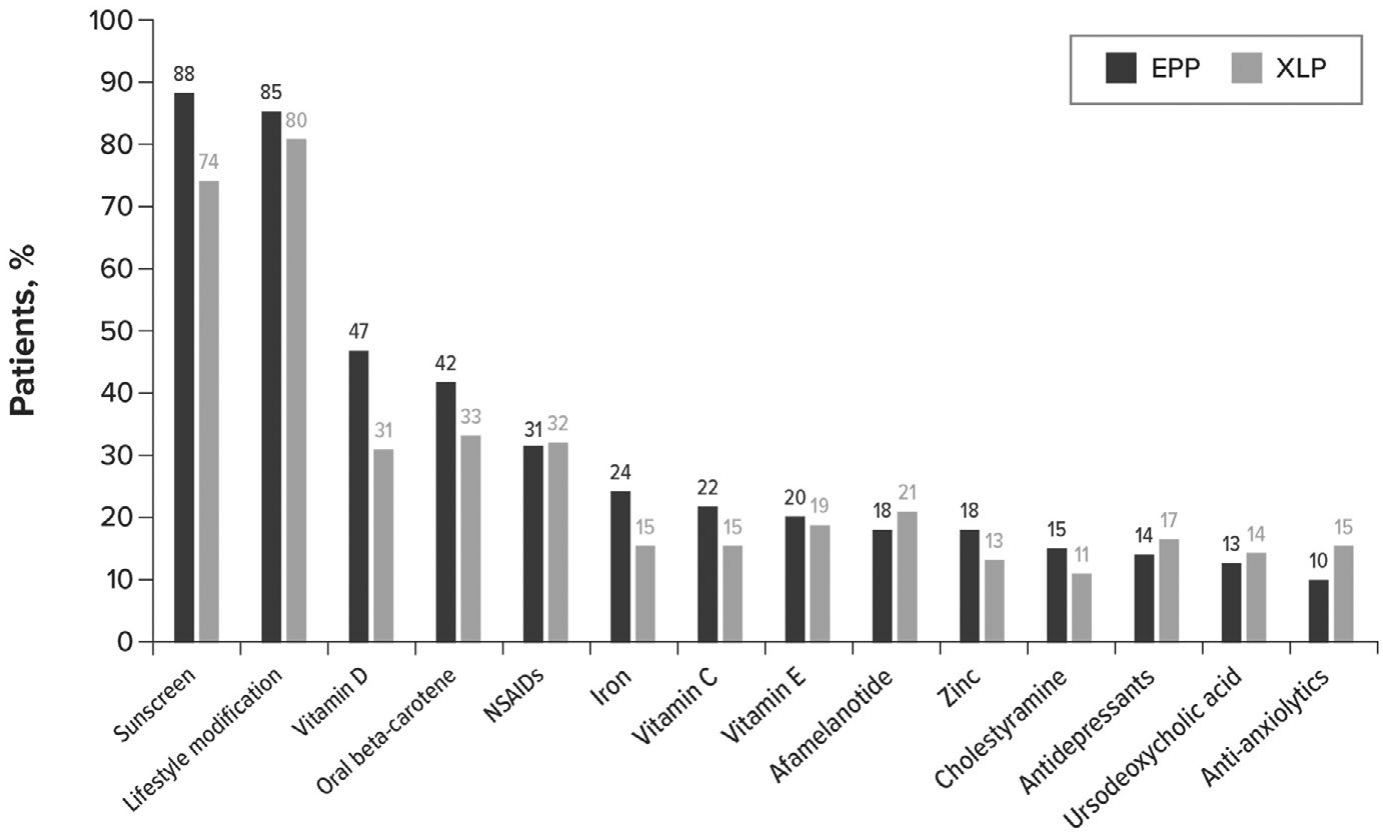
Recommendations and therapy upon and following diagnosis of (*n* = 386) erythropoietic protoporphyria (EPP) and X‐linked protoporphyria (XLP). NSAID, non‐steroidal anti‐inflammatory drug.

Before diagnosis, patients had received a mean (SD) of 4.4 (2.9) recommendations or therapy orders. Upon and following diagnosis, patients received a mean of 5.5 (3.4) recommendations or therapy orders. Primarily, patients were advised to prevent phototoxic reactions by using sunscreen or modifying their lifestyle (Figure 3).

### 3.4 EPP‐/XLP‐related healthcare resource utilization

Within 12 months after diagnosis, patients had a mean (SD) of 4.0 (3.5) office visits and consultations with a median of 3.0, primarily for routine follow‐up (94%), and 2.1 (2.7) specialist referrals with a median of 1.0, primarily to dermatologists (48%) and hematologists (22%).

Between the first report of symptoms in the medical records up to 12 months after diagnosis, 132 patients (34%) had visited an ED due to EPP/XLP, with a mean (SD) number of visits of 0.8 (1.6) and a median of 0. A total of 60 patients (16%) had been hospitalized due to EPP/XLP, with a mean (SD) hospitalization duration of 7.6 (4.5) days and a median of 7.0 days.

### 3.5 EPP‐/XLP‐related cost

Table 3 presents the derived mean and median per‐patient costs. Both the diagnostic pathway costs and the post‐diagnosis tests and referral cost were driven primarily by referrals to specialists, with dermatology referrals making up the majority of costs (mean [SD] USD109.40 [USD137.55] before diagnosis with a median of USD118.62; and USD81.74 [USD110.27] within 12 months of diagnosis with a median of USD0). Inpatient hospitalizations and ED visits between first symptom report and 12 months following diagnosis cost USD2731.85 on average, but most patients did not incur this cost, as reflected by a median cost of USD0. The total per‐patient cost of prescription therapy was driven by a small number of patients, as reflected by a median per‐patient prescription cost of USD192.66.

**TABLE 3:** EPP‐/XLP‐related unadjusted overall per‐patient costs (USD).

Resource	Patients using the resource, *n* (%)	Calculated per‐patient cost	
		Mean (SD)	Median (Q1, Q3)
Total diagnostic pathway	377 (98)	601.54 (518.50)	511.86 (288.96, 770.37)
Specialist referrals	279 (72)	330.64 (432.35)	237.24 (0.00, 355.86)
Diagnostic tests	372 (96)	270.90 (199.33)	314.24 (60.08, 376.23)
Post‐diagnosis tests and referrals	343 (89)	397.68 (432.02)	283.69 (98.87, 558.52)
Specialist referrals	244 (63)	244.21 (320.57)	118.62 (0.00, 355.86)
Diagnostic tests	325 (84)	153.47 (270.81)	153.47 (270.81)
Hospitalizations and ED encounters	141 (37)	2731.85 (7270.04)	0.00 (0.00, 953.72)
Prescription therapies and procedures	263 (68)	27177.59 (109181.97)	192.66 (0.00, 6050.54)
Afamelanotide	68 (18)	25400.51 (108806.29)	0.00 (0.00, 0.00)
Hematin	26 (7)	1061.15 (7418.88)	0.00 (0.00, 0.00)
Cholestyramine	50 (13)	121.66 (807.41)	0.00 (0.00, 0.00)
Ursodeoxycholic acid	43 (11)	126.41 (730.83)	0.00 (0.00, 0.00)
Antidepressant	54 (14)	103.66 (692.71)	0.00 (0.00, 0.00)
Activated charcoal	7 (2)	74.11 (1115.28)	0.00 (0.00, 0.00)
Liver transplant	5 (1.3)	71.71 (626.82)	0.00 (0.00, 0.00)
NSAID	49 (13)	83.96 (88.98)	0.00 (0.00, 0.00)
Anti‐anxiolytic	39 (10)	67.17 (349.44)	0.00 (0.00, 0.00)
Plasmapheresis	16 (4.1)	24.62 (118.52)	0.00 (0.00, 0.00)

Abbreviations: ED, emergency department; EPP, erythropoietic protoporphyria; NSAID, non‐steroidal anti‐inflammatory drug; Q1, first quartile; Q3, third quartile; SD, standard deviation; XLP, X‐linked protoporphyria.

## 4 DISCUSSION

EPP and XLP are rare disorders in which few HCPs have expertise. Guidelines for the diagnosis and management of EPP and XLP were recently published to promote standardized care.^16^ The current study aimed to add to the body of evidence by generating real‐world data regarding the diagnosis and management of EPP and XLP in the US.

Clinicians participating in this retrospective observational study were primarily dermatologists or primary care physicians and were required to have access to medical record data beginning at the onset of EPP or XLP symptoms. These clinicians reported that patients first began experiencing symptoms of EPP or XLP at a mean age of 19 years (median 16 years). However, previous reports from patients, caregivers, and specialist centers indicate that symptoms begin in early childhood.^10^, ^17^ Moreover, clinicians in our study reported that patients generally experience symptoms between 1 and 5 years before diagnosis, whereas earlier studies found this diagnostic delay to be much longer.^10^ Our findings may indicate that clinicians without specialist knowledge of EPP or XLP are not assessing and documenting a full symptom history (e.g., asking patients to recall experiences during summer in childhood). Similarly, 4 patients were reported to have a diagnosis of both EPP and XLP, and a large proportion of patients diagnosed with XLP were female, both of which are highly unlikely characteristics.^18^, ^19^ These findings support the opinion of clinicians who have suggested that increased awareness of these diseases is required.^20^

Diagnosis of EPP or XLP requires testing total erythrocyte protoporphyrin concentration, including proportions of metal‐free and zinc‐bound protoporphyrin.^16^ However, only two‐thirds of patients in our sample received this test before diagnosis. Moreover, not all laboratories can accurately fractionate metal‐free and zinc‐bound protoporphyrins, which can lead to misdiagnosis. Where elevated protoporphyrin is observed, it is recommended to test the *FECH* and *ALAS2* genes to distinguish between EPP and XLP. This genetic test was conducted among 59% of our sample. Testing for urinary and fecal porphyrins is not recommended because of the low sensitivity of this type of analysis; however, these tests were conducted among 42% and 27% of our sample, respectively.

Patients in this study experienced various symptoms; blistering was reported among a third of the sample; however, the consensus among the medical community is that EPP and XLP present as non‐blistering phototoxicity.^6^, ^16^ Guideline‐recommended pharmacological management of EPP symptoms among adults is currently restricted to afamelanotide; however, this therapy is not indicated for pediatric patients or patients with XLP, and there are limitations to access to this drug because administration is restricted to specific centers. There is a clear need for additional efficacious and accessible treatment options. Following diagnosis, most patients were advised to prevent phototoxic reactions by avoidance of sun exposure and use of sunscreen; 40% were prescribed beta‐carotene despite insufficient data regarding its efficacy. Further, despite studies showing that non‐steroidal anti‐inflammatory drugs provide little symptom relief, a third of the sample received recommendations for these drugs for EPP or XLP.

Average costs from symptom onset to diagnosis of EPP or XLP were approximately USD600 per patient. Once diagnosed, average costs of referrals and tests in the first year were approximately USD400 across all patients. Inpatient hospitalizations and ED encounters specifically related to EPP or XLP were seen in fewer than 40% of patients. However, these encounters were costly, resulting in a mean cost of approximately USD2700 per patient. Before diagnosis, two‐thirds of the sample received a prescription for EPP or XLP symptoms, with oral beta‐carotene (27%) and afamelanotide (18%) being the most common. Total average costs of prescriptions and procedures from diagnosis of EPP or XLP to date of last available medical record were over USD27 000 across all patients. However, the resource‐use data were highly skewed (i.e., some patients had very little resource use, while others had multiple uses). Costs for prescriptions and procedures were determined based on the average wholesale price paid by providers. This is not necessarily a reflection of the actual price paid to wholesalers and does not reflect insurance costs or out‐of‐pocket costs to the patient. Thus, caution is advised when interpreting these results.

This study had several limitations, including its retrospective, observational, non‐interventional design in which HCPs were not selected on the basis of their specialist knowledge of EPP or XLP. Therefore, although we consider the findings representative of real‐world clinical practice in the US, HCPs selected for inclusion represented a convenience sample, and, as such, study findings may have limited generalizability to the population of patients treated for EPP and XLP in the US. Data captured were limited to information available in the medical records to which the HCP had access, and data regarding resource use may not reflect the patient's full usage. Although data checks were in place to assess internal consistency of the entered data, responses were not validated by an independent reviewer against the patients' medical records, and thus, neither inter‐ nor intrareliability of the data were assessed. HCPs self‐confirmed patient diagnosis based on the medical record but were not asked to provide evidence of diagnosis. The increased percentage of female patients with XLP and patients scored as having both EPP and XLP made diagnostic certainty problematic. Thus, the sample may capture patients who were misdiagnosed. The median age at which clinicians reported symptom onset was much higher than previously documented reports, which may be indicative of the HCPs not having access to full symptom history information. Finally, costs for referrals and tests were derived from Medicare payments under the Physician Fee Schedule and do not reflect commercial insurance costs or out‐of‐pocket costs to the patient.

This study indicates that patients and HCPs may benefit from education towards increasing their awareness of EPP and XLP. These disorders are associated with significant healthcare costs and require multiple specialist referrals and testing. Patients with EPP and XLP have several unmet needs, including timely and accurate diagnosis with appropriate tests, symptom relief, and efficacious prevention of phototoxic reactions in patients younger than 18 years.

## CONFLICT OF INTEREST STATEMENT

Samuel M. Silver was a paid consultant for Mitsubishi Tanabe Pharma America, Inc. Katherine Houghton, Abby Hitchens, and Valérie Derrien Ansquer are employees of RTI Health Solutions, which received funding to conduct this study. Malgorzata Ciepielewska is an employee of Mitsubishi Tanabe Pharma America, Inc.
